# Screening for Perinatal OCD: A Comparison of the DOCS and the EPDS

**DOI:** 10.1177/10731911211063223

**Published:** 2021-12-30

**Authors:** Nichole Fairbrother, Arianne Albert, Cora Keeney, Devan Tchir, Rose B. Cameron

**Affiliations:** 1 The University of British Columbia, Vancouver, British Columbia, Canada; 2 Women’s Health Research Institute, Vancouver, British Columbia, Canada

**Keywords:** obsessive-compulsive disorder, perinatal mental health, screening

## Abstract

Screening for perinatal-occurring obsessive-compulsive disorder (OCD) is rare. We sought to evaluate the Dimensional Obsessive-Compulsive Scale (DOCS) as a screening tool for perinatal OCD and compare the screening accuracy of the DOCS with the commonly recommended Edinburgh Postnatal Depression Scale (EPDS). English-speaking, pregnant individuals aged 19+ (*N* = 574) completed online questionnaires and diagnostic interviews to assess for OCD prenatally and twice postpartum. The DOCS total score demonstrated the highest level of accuracy. Neither the EPDS-Full nor the three-item Anxiety subscale of the EPDS (EPDS-3A) met the criteria of a sufficiently accurate screening tool for OCD at any of the assessment points. Findings provide support for the DOCS as a screening tool for perinatal OCD and indicate a need for disorder-specific screening for perinatal anxiety and their related disorders (AD). Generalizability of findings is limited to Canada only. Future research would benefit from comparisons with measures of perinatal OCD (e.g., the Perinatal Obsessive-Compulsive Scale).

Obsessive-compulsive disorder (OCD) is an anxiety-related mental health disorder marked by obsessions and compulsions (Coluccia et al., 2016; Stasik et al., 2012). Obsessions are recurrent, unwanted, and distressing thoughts, images, or impulses. Compulsions are repetitive mental or behavioral acts that the person engages in, in an effort to cope with the obsessions. Obsessions typically involve content related to contamination, violence, sex, religion, and/or being responsible for harm to others (Handelzalts et al., 2012). Compulsive behaviors connected to obsessions often involve checking, reassurance seeking, washing and cleaning rituals, and repetitive mental acts (e.g., mentally undoing a negative thought or image). Avoidance of situations related to one’s obsessions is also common.

Frequently, those affected by OCD experience heightened levels of distress and impairment due to their obsessions and/or compulsions (Monk, 2001; Nerum et al., 2013; Newham et al., 2014; Nieminen et al., 2009, 2016). OCD is associated with impaired social functioning, marital difficulties, family relationship problems, increased health care utilization, compromised work functioning, financial problems (Nieminen et al., 2016), and poorer quality of life (Monk, 2001; Nerum et al., 2013; Newham et al., 2014; Nieminen et al., 2009, 2016). As obsessions are often perceived as socially unacceptable to OCD sufferers, they often conceal their obsessions from others, including health care providers (Challacombe & Wroe, 2013), thus impeding access to effective interventions.

Consistent data from individual investigations and a recent meta-analysis indicate an increased risk of OCD during the perinatal period (Fawcett et al., 2019; Ruscio et al., 2010; Russell et al., 2013). Indeed, one in seven perinatal people (15%) experience OCD at some point during pregnancy or the first 6 months postpartum (Fairbrother et al., 2021). This increased prevalence has been found to peak in the weeks following childbirth, after which it declines (Brok et al., 2017; Fairbrother et al., 2021). When OCD occurs postpartum, it is frequently characterized by obsessions of harm related to the newborn (Fairbrother & Abramowitz, 2016), specifically thoughts and images of harming (intentionally or accidentally) the infant. Overt compulsions are less common in postpartum OCD (ppOCD) compared with non-perinatal OCD. However, avoidance of the infant or avoidance of specific activities with one’s infant (e.g., bathing the infant or using knives near the infant) is common. When compulsions are present, they are often in the form of checking (e.g., checking on the infant’s breathing or checking the internet for reassurance) and mental compulsions (e.g., mentally undoing the obsession by imagining one’s infant to be well) (Fairbrother & Abramowitz, 2016; Russell et al., 2013).

For perinatal people, OCD has negative implications for fetal and newborn health, parenting, and infant development (Saisto et al., 2001; Salomonsson et al., 2013; Stoll et al., 2016; Straub et al., 2012; Sydsjö et al., 2014, 2015; Takegata et al., 2015). The consequences of ppOCD may involve impaired functioning, reduced ability to complete daily tasks, strain on relationships, and negative effects to fetal, newborn, and infant well-being (Brander et al., 2016; Challacombe et al., 2016; Coplan et al., 2005; House et al., 2016; Uguz et al., 2015). In addition, comorbid depressed mood is common among people affected by ppOCD (Miller et al., 2015).

## Screening for Anxiety and Anxiety-Related Disorders Among Perinatal People

Over the past few years, there have been urgent calls from health care agencies around the world (e.g., the United States, Canada, and Australia) for accurate and reliable screening tools for perinatal anxiety and their related disorders (AD). Typically, this includes the core anxiety conditions listed in the *Diagnostic and Statistical Manual of Mental Disorders* (5th ed.; *DSM-5*; American Psychiatric Association, 2013), as well as OCD and posttraumatic stress disorder (PTSD). Despite an urgent need and calls for evidence-based screening measures, the overwhelming consensus among scientists working in this area is that the current evidence is too weak to support recommending any specific tool to screen for perinatal AD. A key difficulty in identifying an accurate and reliable screening tool for perinatal AD has been the methodological quality of the studies conducted. While a large number of studies have evaluated the screening accuracy of various tools, few to none have employed high-quality methodology.

Further, to merit broad implementation, screening tools should meet minimum criteria of accuracy. The empirical literature supports the following minimum criteria be met for a screening tool to be deemed “sufficiently accurate” as to merit implementation (Bossuyt et al., 2015; Cohen et al., 2016; Zimmerman et al., 2004). Specifically, to be recommended for widespread use, a screening tool should demonstrate an area under the curve (AUC) ≥0.8 (≥0.8 is generally considered excellent; Mandrekar, 2010), a Youden’s Index of ≥0.5 (i.e., when sensitivity = 0.75, specificity = ≥0.75), a negative predictive value (NPV) of ≥0.8, and a positive likelihood ratio (LR+) of ≥4.0. An LR+ of 4.0 means that with a positive test result, the probability the person has the disease increases 25% over pretest probability (Sackett et al., 2000).

## Using the Edinburgh Postnatal Depression Scale (EPDS) to Screen for Perinatal OCD

The EPDS (Cox et al., 1987) is a 10-item measure of depressed mood among perinatal period. It is arguably the most commonly used measure of perinatal depression (Matthey et al., 2013). Three items of the EPDS have been identified as measuring anxiety and these three items have been labeled the three-item Anxiety subscale of the EPDS (EPDS-3A; Matthey, 2008; Matthey et al., 2013). Both the 10-item EPDS-Full and the EPDS-3A have been evaluated as screening tools for both perinatal depression and perinatal anxiety and anxiety-related disorders (Cox et al., 1987; Grigoriadis et al., 2011). To date, all studies of the EPDS/EPDS-3A as screening tools for the anxiety and their related conditions have evaluated these conditions as a group and not individually (Fairbrother et al., 2019; van Heyningen et al., 2018).

Only four studies have assessed the EPDS as a screening tool for perinatal AD employing some Gold Standard methodology. Of these, all four evaluated the EPDS-3A and one the EPDS-Full version. In the one study to assess the EPDS-Full version, it failed to meet the criteria of a sufficiently accurate screening tool. Among the four studies to assess the EPDS-3A, only one demonstrated an AUC of greater than 0.80. Although NPV values were sufficient across the three studies that reported them, in none of the four studies was the *J* index ≥0.50.

While there is an urgent need to identify accurate and valid screening tools for perinatal AD as a whole (Massachusetts General Hospital Centre for Women’s Mental Health, 2019), it is equally important to know how well they perform in detecting individual disorders, in particular those with low prevalence. It is possible for a screening tool to perform well for perinatal AD overall and yet neglect one or more low prevalence conditions, consequently neglecting to detect those with these difficulties. Should the EPDS-3A prove to be an accurate screening tool for perinatal anxiety, this would have significant practical implications: as the EPDS is currently in widespread use as a screening tool for perinatal depression, implementation of the EPDS requires additional scoring only but not additional administration. Consequently, motivation to evaluate the EPDS-3A as a potential screening tool for perinatal AD is high. In addition to appearing to perform poorly, the EPDS-3A has yet to be evaluated as a screening tool specifically for perinatal OCD.

## Screening for OCD

The majority of studies evaluating the screening accuracy of self-report measures of OCD in adults have assessed the Obsessive-Compulsive Inventory–Revised (OCI-R; Abramowitz & Deacon, 2006; Abramowitz et al., 2010; Foa et al., 2002; Gönner et al., 2007; Tang et al., 2015; Williams et al., 2013; Wootton et al., 2015), and the Dimensional Obsessive-Compulsive Scale (DOCS; Abramowitz et al., 2010; López-Solà et al., 2014; Thibodeau et al., 2015) or the DOCS–Short Form (DOCS-SF; Eilertsen et al., 2017). While both the OCI-R and the DOCS have been documented as possessing strong screening metrics, available data indicate that the DOCS (including the DOCS-SF) is the more accurate of the two (Abramowitz et al., 2010; Eilertsen et al., 2017; López-Solà et al., 2014; Rapp et al., 2016). To date, only the Perinatal Obsessive-Compulsive Scale (POCS) and the Yale Brown Obsessive-Compulsive Scale–Self-Report (Y-BOCS self-report) have been evaluated as potential screening tools for perinatal OCD (Lord et al., 2011). The POCS resulted in stronger screening metrics (i.e., AUC = 0.81, *J* = 0.78) compared with the Y-BOCS (i.e., AUC = 0.75, *J* = 0.73). The screening accuracy of the DOCS has yet to be evaluated.

The purpose of this study was to evaluate the DOCS as screening tool for perinatal OCD and to compare the accuracy of the DOCS with (a) the criteria for a “sufficiently accurate” measure and (b) the accuracy of the EPDS and the EPDS-3A in screening for OCD among perinatal people. We hypothesized that disorder-specific screening for OCD (i.e., the DOCS) would result in superior accuracy to generic screening for OCD (i.e., the EPDS-Full and EPDS-3A), both in pregnancy and the postpartum, and that disorder-specific screening may be needed to achieve the standard of a sufficiently accurate measure.

## Method

This research was conducted as part of a larger study, for which a detailed study protocol has been published (Collardeau et al., 2019).

### Ethics

This province-wide study was approved by the University of British Columbia Behavioral Research Ethics Board, the Island Health, Health Research Ethics Board, the Fraser Health Research Ethics Board, and Vancouver Coastal Health. Due to the sensitive nature of some aspects of the broader study, written consent was obtained at both the initial prenatal assessment and the first postpartum assessment. Oral consent was also provided at the beginning of each study interview. A study debriefing letter was provided to all participants upon completion of participation.

### Participants

All English-speaking, pregnant individuals over the age of 19 and living in the Canadian Province of British Columbia (BC) were eligible to take part in this study. In total, 763 individuals participated. A total of 574 of these contributed data to the current study. Data collection occurred from February 9, 2014 until February 14, 2017.

### Recruitment

To promote sample representativeness, we employed a variety of recruitment strategies, including hospital-based recruitment (85.3%), community-based recruitment (13.3%), and approaches focused specifically on rural areas of BC (1.4%). For hospital-based recruitment, we recruited proportionally across hospitals in BC in which 1,500 or more live births occur annually and used primarily direct approach methods (i.e., approaching pregnant individuals waiting for routine antenatal appointments). Additional participants were recruited using direct and/or indirect recruitment methods at private clinics, trade shows, community events, and prenatal centers throughout BC. Individuals who expressed an interest via telephone, email, or in-person, and met the study eligibility requirements, were invited to participate.

### Procedures

Participants were followed from the third trimester of pregnancy (at the earliest 32 weeks’ gestation) to a maximum of 9 months postpartum. They were asked to complete online questionnaires followed by a telephone interview at three separate timepoints—once in late pregnancy (*M* = 36.89 weeks, *SD* = 1.96) and twice postpartum (*M* = 9.09 weeks, *SD* = 1.94; and *M* = 21.27 weeks, *SD* = 3.83). Questionnaires were primarily completed online; however, if necessary, participants could choose to complete questionnaires via paper hardcopy sent to their home to be returned by mail. Participants who did not complete a questionnaire and/or interview at earlier timepoints were nevertheless eligible to complete the assessments at subsequent stages.

Not all 574 participants provided data for all three assessment points (e.g., some participants may have completed all three interviews but missed one of the questionnaire assessments). Of the 574 participants who contributed data to the present inquiry, 573 provided data for the prenatal assessment, 542 for the early postpartum assessment, and 394 for the late postpartum assessment.

### Measures

Demographic (age, marital status, occupation, education, income, race/ethnicity, and language), pregnancy (medical and pregnancy complications, and reproductive history), and birth (baby’s date of birth, mode and location of delivery, birth weight, pregnancy and birth complications, neonatal health, and infant feeding) information was collected via self-report.

The DOCS (Abramowitz et al., 2010) is a 20-item self-report measure used to assess the four most consistently replicated OCD symptoms, using a four-factor model corresponding to the measure’s subscales: (a) Germs and Contamination; (b) Responsibility for Harm, Injury, or Mistakes; (c) Unacceptable Obsessional Thoughts; and (d) Symmetry, Completeness, and Ordering. Five items (rated 0–4) assess the parameters of severity of time occupied by obsessions and rituals, avoidance, distress, functional interference, and difficulty disregarding the obsessive thoughts and refraining from the compulsions within each symptom dimension (Abramowitz et al., 2010; Enander et al., 2012; Thibodeau et al., 2015). DOCS subscales have excellent test-retest reliability in clinical samples (α = .87–.96) and in student samples (α = .82–.93) as well as strong convergent, discriminant, and construct validity (Abramowitz et al., 2010; Enander et al., 2012; Thibodeau et al., 2015). The measure is sensitive to changes over time and incremental increases on the DOCS represent actual increases in OCD symptoms (Thibodeau et al., 2015). Evidence suggests the DOCS is accurate in detecting OCD from healthy controls, with AUCs consistently exceeding 0.8 (Abramowitz et al., 2010; Eilertsen et al., 2017; López-Solà et al., 2014; Thibodeau et al., 2015).

The EPDS (Cox et al., 1987) is a self-report measure widely used to screen for postnatal depression (Jomeen & Martin, 2005). The EPDS contains 10 items with four response options each rated 0 to 3. As it has been developed for use in postpartum samples, it de-emphasizes the somatic symptoms that may overlap with depressive symptoms but would be considered normative during this period (Gaynes et al., 2005). It has demonstrated good to excellent psychometric properties and acceptable ranges of sensitivity and specificity (70%–100% for sensitivity and 74%–97% for specificity in the antenatal period; 65%–100% for sensitivity and 49%–100% for specificity in the postnatal period; Kozinszky & Dudas, 2015). Factor analytic studies of the EPDS support a distinct three-item Anxiety subscale (EPDS-3A; Matthey, 2008). Both the EPDS and the EPDS-3A have acceptable internal consistency reliability among perinatal people (Swalm et al., 2010) and the EPDS-3A is effective in screening for perinatal anxiety (Fairbrother et al., 2019). Literature assessing EPDS-3A with sufficient methodological criteria indicates moderate accuracy; Fairbrother et al. (2019) report an AUC of 0.76, and Van Heyningen and others (2018) report an AUC of 0.69.

### Diagnostic Interviews

The *Structured Clinical Interview for DSM-5* (SCID-5; First et al., 2016) is a well-validated, semi-structured interview for *DSM-5* diagnoses. Interviewers were trained to strict criteria by the principal investigator. Symptom severity was rated from 0 (*none*) to 8 (*very severe/disabling*), with “0” representing a diagnostic status of absent or in full remission, “0.5–2.5” representing partial remission, “3.0–3.5” representing subclinical diagnoses, and “4.0–8.0” representing the range of diagnostic severity for those whose symptoms met full diagnostic criteria.

At each interview, participants were asked about obsessive-compulsive (OC) symptoms experienced in the 2 weeks prior. In addition, in all but the first postpartum interview, participants were asked to identify the 2-week period (prenatal or postpartum) during which their OC symptoms were most intense. This permitted an assessment of prenatal and postpartum prevalence (reported in a separate publication; Fairbrother et al., 2021). Diagnostic status and diagnostic severity ratings were specified for both current and most intense time periods. In addition to standard diagnostic questions, during the postpartum interviews, participants were also asked about any thoughts of infant-related harm (both accidental and intentional harm) and associated behaviors. The overall evaluation of OCD diagnostic status and severity included evaluations of obsessions and compulsions of infant-related harm.

Upon completion of data collection, reliability checks were undertaken by a senior interviewer and two external OCD specialists. A quarter of all audio-recorded interviews with individuals diagnosed with significant OCD symptomatology (subclinical, clinical, partial remission) were reviewed for reliability, along with 5% of interviews in which no diagnosis of OCD was reported. Interviews from each of the 10 interviewers were proportionally and randomly sampled at each timepoint. Interrater reliability among the three raters was rated as good to excellent using an intraclass correlation coefficient with a two-way random effect model for consistency, with scores ranging from .75 to .98 across the three timepoints.

### Statistical Analyses

Analyses were carried out using SPSS 2017 (IBM Corp., 2017) and R software (R Core Team, 2020). Descriptive statistics are presented as means, standard deviations, and correlations. Receiver operating characteristic (ROC) curves were constructed for all DOCS subscales, the total DOCS scores, the EPDS, and the EPDS-3A. ROC curves were constructed using the “cutpointr” package (Thiele, 2020) in R (R Core Team, 2020). The optimal cutpoint was estimated by maximizing Youden’s index over 5,000 bootstrap replicates at each timepoint for each subscale. OCD was defined as meeting full diagnostic criteria. The number of completed measures differs across assessment points.

## Results

### Participants

Participant demographic and reproductive data are presented in Table 1. Participants in this study differed somewhat from the larger study population in terms of parity, χ^2^(1, *N* = 133,636) = 26.9, *p* < .001; mode of delivery, χ^2^(1, *N* = 133,600) = 6.2, *p* = .01; and age, χ^2^(6, *N* = 133,637) = 28.5, *p* < .001. Specifically, when compared with provincial data, our sample contained a greater proportion of people who had never given birth (55% vs. 46%), were slightly older (63% 30- to 40-year-olds vs. 58%), and gave birth via Cesarian section (35% vs. 33%). Eleven (3.6%) met full criteria for OCD in pregnancy, 43 (10.6%) at 2 months postpartum, and 22 (5.8%) at 5 months postpartum.

**Table 1.** Demographic Information, Reproductive History, and Medical and Pregnancy Complications (N = 574).

Demographic characteristics	% of total sample
Relationship status	
Married or living with a romantic partner	95.3
Single	3.6
Divorced/separated	1.1
Education	
Did not complete high school	2.0
Completed high school	7.1
Some undergraduate education	51.1
Some graduate education	39.8
Cultural heritage	
European	56.5
East Asian	11.5
South Asian	6.2
Southeast Asian	5.6
Indigenous	2.7
Other	17.4
Age in years	*M* = 32.5 (*SD* = 4.9), range = 18–47
Reproductive history	% of total sample
First pregnancy	39.9
Prior history of miscarriage	25.5
Prior history of late loss	1.3
Primiparous	69.9
Current pregnancy and birth	% of total sample
Mode of delivery	
Vaginal	62.1
Cesarean (before the onset of labor)	37.9
Complications during labor	30.5
Episiotomy performed	9.1
Readmission to the hospital (parent who carried pregnancy)	8.1
Baby admitted to intensive or special care unit	12.3

### ROC Curves and Diagnostic Accuracy

**Figure 1.**
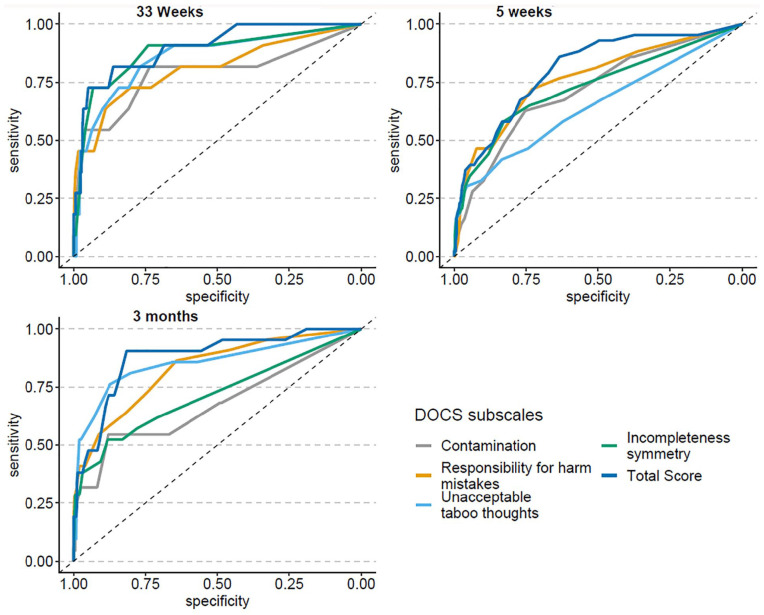
ROC Curves for DOCS Total and Subscale Scores. *Note.* ROC = Receiver Operating Characteristic; DOCS = Dimensional Obsessive-Compulsive Scale.

**Figure 2.**
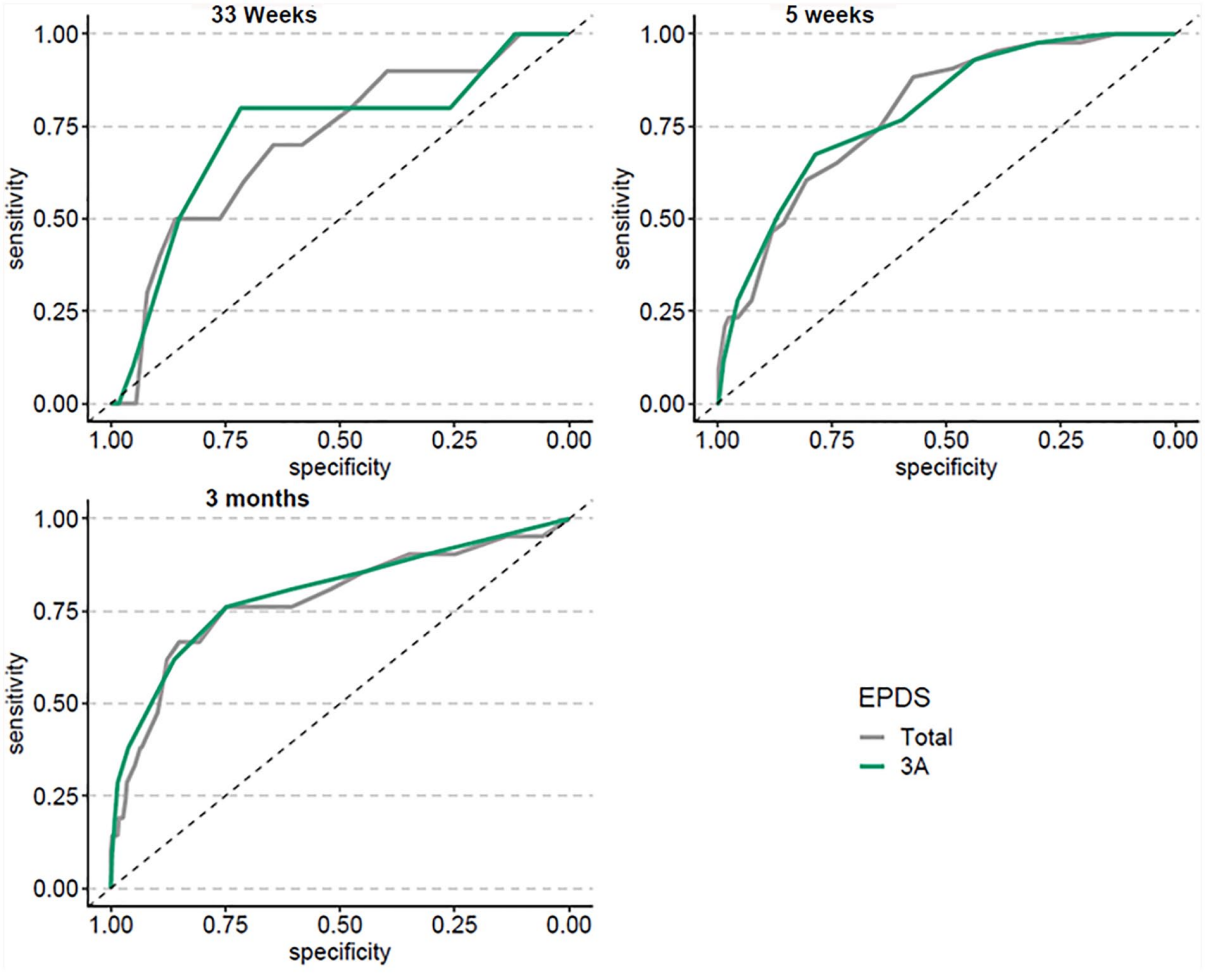
ROC Curves for EPDS-Full and EPDS-3A Scores. *Note.* ROC = Receiver Operating Characteristic; EPDS = Edinburgh Postnatal Depression Scale; EPDS-3A = three-item Anxiety subscale of the EPDS.

ROC curves for the DOCS (the four subscales and the total score) and the EPDS-Full and EPDS-3A are shown in Figures 1 and 2, respectively. Means and standard deviations for scales and subscales are listed in Table 2, and indices of diagnostic accuracy are shown in Table 3 (for the DOCS) and Table 4 (for the EPDS-Full and the EPDS-3A). ROC curves show the AUC in pregnancy and at approximately 2 and 5 months postpartum, for each of the DOCS subscales and the DOCS total score (Figure 1) as well as the EPDS-Full and the EPDS-3A (Figure 2). AUC values ranged from 0.65 to 0.88 for the individual DOCS subscales, and from 0.81 to 0.90 for the full scale. For the EPDS, AUC values ranged from 0.71 to 0.79 for the EPDS-Full, and from 0.73 to 0.80 for the EPDS-3A.

**Table 2.** Means and Standard Deviations for Scales and Subscales.

DOCS and EPDS Totals and Subscales	Prenatal (*n* = 573) *M* (*SD*)	Early Postpartum (*n* = 542) *M* (*SD*)	Late Postpartum (*n* = 394) *M* (*SD*)
DOCS Contamination	2.85 (2.90)	2.72 (2.76)	2.08 (2.56)
DOCS Responsibility/Harm	2.74 (2.91)	2.72 (2.98)	2.46 (2.50)
DOCS Unacceptable Thoughts	2.13 (3.00)	1.96 (2.75)	1.56 (2.29)
DOCS Incompleteness/Symmetry	2.02 (2.87)	1.95 (2.89)	1.22 (2.27)
DOCS Total	9.81 (9.46)	9.33 (9.28)	7.31 (7.74)
EPDS-Full	7.15 (4.81)	7.00 (4.67)	6.36 (4.81)
EPDS-3A	3.38 (2.14)	3.23 (2.15)	3.09 (2.18)

*Note.* DOCS = Dimensional Obsessive-Compulsive Scale; EPDS = Edinburgh Postnatal Depression Scale; EPDS-3A = three-item Anxiety subscale of the EPDS.

**Table 3.** DOCS ROC Analysis Data.

DOCS Total and Subscales by Assessment Point	AUC [95% CI]	*J*	Cutpoint	Sensitivity	Specificity	PPV	NPV	LR+
DOCS Contamination								
Prenatal	0.79 [0.60, 0.97]	0.42	5.41	0.55	0.88	0.14	0.98	4.58
Early postpartum	0.72 [0.63, 0.80]	0.38	3.56	0.63	0.75	0.23	0.94	2.52
Late postpartum	0.68 [0.54, 0.82]	0.42	4.70	0.55	0.88	0.22	0.97	4.58
DOCS Responsibility/Harm								
Prenatal	0.81 [0.64, 0.98]	0.53	5.34	0.64	0.89	0.18	0.99	5.82
Early postpartum	0.77 [0.68, 0.85]	0.45	3.96	0.72	0.73	0.24	0.96	2.67
Late postpartum	0.83 [0.73, 0.93]	0.47	3.71	0.73	0.74	0.15	0.98	2.81
DOCS Unacceptable Thoughts								
Prenatal	0.86 [0.72, 0.99]	0.54	3.74	0.73	0.81	0.12	0.99	3.84
Early postpartum	0.65 [0.55, 0.74]	0.23	4.43	0.33	0.91	0.30	0.92	3.67
Late postpartum	0.85 [0.75, 0.96]	0.63	3.46	0.76	0.87	0.26	0.98	5.85
DOCS Incompleteness/Symmetry								
Late prenatal	0.88 [0.75, 1.0]	0.61	4.25	0.73	0.88	0.18	0.99	6.08
Early postpartum	0.73 [0.64, 0.82]	0.41	3.20	0.58	0.83	0.29	0.94	3.41
Late postpartum	0.72 [0.56, 0.84]	0.40	3.66	0.52	0.88	0.20	0.97	4.33
DOCS Total								
Late prenatal	0.90 [0.80, 1.0]	0.64	18.60	0.73	0.91	0.23	0.99	8.11
Early postpartum	0.81 [0.74, 0.88]	0.46	8.84	0.79	0.67	0.22	0.96	2.39
Late postpartum	0.88 [0.80, 0.96]	0.72	10.82	0.90	0.82	0.23	0.99	5.00

*Note.* DOCS = Dimensional Obsessive-Compulsive Scale; ROC = receiver operating characteristic; AUC = area under the curve; CI = confidence interval; LR+ = positive likelihood ratio; PPV = p﻿ositive p﻿redictive v﻿alue; NPV = n﻿egative p﻿redictive v﻿alue.

**Table 4.** EPDS and EPDS-3A ROC Analysis Data.

EPDS-Full and 3A by Assessment Point	AUC [95% CI]	*J*	Cutpoint	Sensitivity	Specificity	PPV	NPV	LR+
EPDS-Full								
Prenatal	0.71 [0.54, 0.88]	0.26	9.05	0.50	0.76	0.07	0.98	2.08
Early postpartum	0.79 [0.72, 0.85]	0.39	7.43	0.74	0.65	0.20	0.95	2.11
Late postpartum	0.78 [0.66, 0.90]	0.52	10.01	0.67	0.85	0.22	0.98	4.47
EPDS-3A								
Prenatal	0.73 [0.55, 0.91]	0.52	4.63	0.80	0.72	0.09	0.99	2.86
Early postpartum	0.79 [0.72, 0.86]	0.46	4.35	0.67	0.79	0.28	0.95	3.19
Late postpartum	0.80 [0.68, 0.92]	0.51	4.90	0.76	0.75	0.16	0.98	3.04

*Note.* EPDS = Edinburgh Postnatal Depression Scale; EPDS-3A = three-item Anxiety subscale of the EPDS; ROC = receiver operating characteristic; AUC = area under the curve; CI = confidence interval; LR+ = positive likelihood ratio; PPV = p﻿ositive p﻿redictive v﻿alue; NPV = n﻿egative p﻿redictive v﻿alue.

## Discussion

In this study of a representative sample of perinatal Canadians, we sought to assess and compare the accuracy of the DOCS and the EPDS (Full and 3A) as screening tools for perinatal OCD. The screening accuracy metrics of each of the evaluated measures were compared with the criteria for a “sufficiently accurate” measure.

The DOCS demonstrated a very high level of screening accuracy, significantly exceeding the criteria for a “sufficiently accurate” measure, at one or more assessment points, for three of the four subscales, and the DOCS total scores. Overall, the DOCS total score demonstrated the highest level of performance, with screening metrics mirroring those found in other, non-perinatal assessments of the DOCS as a screening tool for OCD (Abramowitz et al., 2010; Eilertsen et al., 2017; López-Solà et al., 2014; Thibodeau et al., 2015).

Of the four DOCS subscales, the Unacceptable Thoughts subscale demonstrated the highest level of accuracy. Specifically, at the late postpartum assessment, the Unacceptable Thoughts subscale exceeded the criteria for a “sufficiently accurate” measure across all three metrics (AUC = 0.85, *J* = 0.63, LR+ = 5.85), and at the prenatal assessment, it exceeded the criteria on two out of the three metrics (AUC = 0.86, *J* = 0.54, LR+ = 3.84). At the early postpartum assessment, however, it met only one criterion for a “sufficiently accurate” measure (i.e., AUC = 0.81). These findings (i.e., lower accuracy at the early postpartum assessment, and superior performance compared with other DOCS subscales) are consistent with our earlier findings from this research. Specifically, based on diagnostic interviews, we found a very high level of OC symptoms at the early postpartum assessment (Fairbrother et al., 2021), suggesting that some symptoms of OCD in the early postpartum may be a normative postpartum experience. It is probable that the high prevalence of OC symptoms at this assessment point may have diluted the screening accuracy of this subscale. In addition, the majority of participants in our research who met criteria for OCD (in particular at the postpartum assessment) reported obsessions involving unwanted, intrusive thoughts of infant-related harm. This is consistent with the finding that the DOCS Responsibility/Harm and Unacceptable Thoughts subscales demonstrated higher accuracy compared with the Contamination subscale. Finally, the Incompleteness/Symmetry subscale demonstrated a very high level of accuracy but only at the prenatal assessment. It is possible that this reflects the tendency for pregnant people to engage in high levels of “nesting” behavior (e.g., organizing, arranging, and preparing for the infant to arrive; Anderson & Rutherford, 2013).

As predicted, only the DOCS met the criteria of a “sufficiently accurate” measure. At none of the three assessment points did the EPDS-Full nor the EPDS-3A meet the criteria of a “sufficiently accurate” screening tool. The EPDS-3A demonstrated the highest level of accuracy at the late postpartum assessment where it met two of the three criteria for a “sufficiently accurate” measure. As expected, the DOCS (total and some subscales) outperformed both the EPDS-Full and the EPDS-3A as a screening tool for perinatal OCD.

Study findings provide strong evidence that the DOCS provides high accuracy in screening for perinatal OCD. What is also clear from the present findings is that, not surprisingly, disorder-specific measures (e.g., the DOCS) provide more accurate screening outcomes compared with more generic measures (e.g., the EPDS). What was perhaps more surprising was the fact that the EPDS-3A approached the standard of a “sufficiently accurate” screening tool, at least for the late postpartum assessment. The obtained metrics for the EPDS-3A suggest that while not fully sufficient, it is also not altogether inadequate as a screening tool for perinatal OCD. Given the ubiquity of the EPDS as a screening tool for perinatal depression, and more recently the EPDS-3A as a screening tool for perinatal anxiety (Cox et al., 1987; Grigoriadis et al., 2011), this is a welcome discovery.

While preliminary data indicate that the EPDS-3A may perform reasonably well as a screening instrument for perinatal anxiety and related disorders as a whole, it may perform less well for specific disorders and is also unable to provide any information regarding which disorder the person is most likely to be experiencing. A perhaps more promising and, to date, more accurate measure is the Anxiety Disorder-13 scale (AD-13; Fairbrother et al., 2019). If the AD-13 proves to be accurate in screening for individual disorders, and not only the anxiety and related disorders as a group, it has the ability to identify which specific disorders may require a follow-up screening instrument.

A curious aspect of our findings is the fact that all of the scales/subscales performed least well at the time of the early postpartum assessment. We suspect that this is a product of the fact that OC symptoms were highest at this time (Fairbrother et al., 2021) and may not always represent difficulties likely to persist. It is now well documented that unwanted, intrusive, infant-related thoughts, images, and impulses are ubiquitous in the early postpartum but tend to decrease later in the postpartum. It is likely that some OC symptoms in the early postpartum represent a normal postpartum phenomenon rather than any underlying psychopathology (Fairbrother & Woody, 2008). This phenomenon may interfere with OCD screening accuracy.

### Limitations

Strengths of the current study include multiple assessments from pregnancy through to 5 months postpartum and the use of high-quality methodology to evaluate screening accuracy. A key limitation is the fact that we did not include a comparison of the DOCS with the POCS. This would have significantly strengthened this work and provided important additional information. This comparison should be undertaken in future research. Moreover, despite a recruitment method carefully designed to promote sample representativeness, data were collected in only one Canadian province, limiting the generalizability of findings to other cultures.

## Conclusion and Clinical Implications

Our data suggest that screening for perinatal OCD may be most beneficial in pregnancy and at 4 to 6 months postpartum. As it can be challenging in health care settings to administer separate screening tools for each disorder of interest, it is very encouraging that both the Unacceptable Thoughts subscale of the DOCS and the EPDS-3A performed reasonably well as screening tools for perinatal OCD, with the DOCS Unacceptable Thoughts subscale outperforming the EPDS-3A. Decisions regarding whether to employ the DOCS full scale, the DOCS unacceptable thoughts, or the EPDS-3A should be made on the basis of available resources, burden on patients, and the objectives in the particular clinical context in which the measure is to be administered.
